# Bi‐Regional Machine Learning Radiomics Based on CT Noninvasively Predicts LOX Expression Level and Overall Survival in Hepatocellular Carcinoma

**DOI:** 10.1002/cam4.71154

**Published:** 2025-08-12

**Authors:** Kexin Gao, Musa Yaermaimaiti, Yiwei Wang, Guanggai Xia, Ting Xu, Hongcheng Wang

**Affiliations:** ^1^ Department of General Surgery Shanghai Sixth People's Hospital Affiliated to Shanghai Jiao Tong University School of Medicine Shanghai China; ^2^ Shenzhen Children's Hospital Shenzhen China; ^3^ Department of General Surgery Kashgar Prefecture Second People's Hospital Kashgar China

**Keywords:** hepatocellular carcinoma, LOX, machine learning, prognosis, radiomics, survival

## Abstract

**Objective:**

We aimed to assess the association between lysyl oxidases (LOX) expression levels and the prognosis of patients with Hepatocellular carcinoma (HCC) and to establish a CT‐based bi‐regional radiomics model that can discriminate LOX expression level using The Cancer Imaging Archive (TCIA) and The Cancer Genome Atlas (TCGA) database.

**Methods:**

294 HCC samples were downloaded from TCGA for gene‐based prognostic analysis. Meanwhile, the underlying molecular mechanism of LOX expression and its relationship with the immune microenvironment was investigated. Thirty‐four cases that had preoperative computed tomography (CT) images stored in TCIA with genomic data in TCGA were used for radiomics feature extraction and model construction. The association of this combined LOX‐based radiomics model with HCC prognosis was evaluated.

**Results:**

The expression of LOX in tumor tissue significantly correlated with overall survival (OS). LOX was involved in the regulation of immune response and tumor invasion and metastasis. Two radiomic models were developed using the least absolute shrinkage and selection operator (LASSO) regression analysis. The model of the whole‐tumor region has a good predicting effect in LOX expression, with the area under the receiver operating characteristic (ROC) curve being 0.775 in the training set, while the average under the curve (AUC) value of the 5‐fold cross‐validation was 0.754. The model of the whole‐tumor and peri‐tumor region also has a good predicting effect in LOX expression, with the area under the ROC curve being 0.800 in the training set, while the average AUC value of the 5‐fold cross‐validation was 0.796. The calibration curves and the Hosmer‐Lemeshow test revealed consistency of the prediction probability acquired by our models and the true value of LOX expression, while the decision curve analysis (DCA) curve showed that both models had clinical practicability.

**Conclusion:**

LOX expression can influence the prognosis of patients with HCC, which can be predicted noninvasively by CT image‐based radiomics.

Hepatocarcinoma is the sixth most common malignant tumor worldwide, which ranks as the third leading cause of cancer‐related deaths. Hepatocellular carcinoma (HCC) accounts for 75%–85% of the primary hepatocarcinoma. Despite remarkable advances in the treatment of liver cancer, such as surgery, chemotherapy, immunotherapy, and targeted therapy, HCC patients have a poor prognosis, with a median survival of 6 to 20 months after diagnosis and a 5‐year survival rate of 10% [1]. HCC is a biologically heterogeneous malignancy with different risks of recurrence and/or metastasis and varying sensitivities to different treatments. HCC confirmed by two similarly sized pathologic analyses may have different outcomes [2]. This poses a major challenge to the prediction of overall survival (OS) and treatment outcome. Classic prognostic indicators, including clinicopathological features, laboratory biomarkers (such as AFP) and imaging examinations (e.g., ultrasound, CT and MRI) are difficult to meet the need for personalized precision treatment [3]. Thus, it is necessary to search for other reliable indicators to evaluate the prognosis of patients with HCC.

The tumor microenvironment has a significant impact on prognosis and clinical response to treatment. As the main part of the tumor microenvironment, the extracellular matrix (ECM) co‐regulates the abnormal expression of the tumor microenvironment with tumor cells and secretes a variety of growth factors, cell factors, and chemokines, ultimately promoting the progression of tumor [4]. The lysyl oxidase (LOX) family is a group of copper‐containing amine oxidases composed of LOX and LOX‐like proteins (LOXL1, LOXL2, LOXL3, and LOXL4), which play a key role in maintaining ECM homeostasis and can maintain the hardness and structural stability of ECM by catalyzing the covalent cross‐linking of collagen and elastin [5]. LOX also participates in the differentiation, malignant transformation, and invasiveness of tumor cells as a classic hypoxia‐induced gene. Besides, LOX is a new macrophage chemokine [6]. It has been reported that LOX is associated with several types of cancer, including breast, colorectal, prostate, gastric, and pancreatic cancer, head and neck squamous cell carcinoma, renal clear cell carcinoma, melanoma, oral and oropharyngeal squamous cell carcinoma, as well as basal and squamous cell skin carcinoma [7], with some more recent studies showing an association between LOX and HCC. High LOX expression is associated with epithelial‐mesenchymal transition (EMT) markers and predicts early recurrence and poor survival in patients with HCC [8]. Zhu J et al. found that TGFB‐mediated VEGF was downregulated in HCC cells, which was influenced by LOX through the p38/MAPK signaling pathway; in addition, LOX knockout in HCC cells suppressed the proliferation, migration, and invasion, and the expression of VEGF was declined [9]. LOX also has a critical role in endothelial cell (EC) proliferation and contributes to angiogenesis in HCC via VEGF expression, which can be induced by tumor initiating cell (TIC) enrichment [10]. Furthermore, Tan HY et al. demonstrated that LOXL4 plays an important role in fostering an immunosuppressive microenvironment during hepatocarcinogenesis [11].

Currently, the expression of LOX can be monitored by detecting chemokines of peripheral blood and mRNA or protein from fresh tissue samples, which can be easily interfered with by the operator, antibodies, and sample collection; although detection based on paraffin sections is an alternative, it is hard to get rid of interference from the operator, antibodies, and the cost. However, our current knowledge suggests that non‐invasive and effective methods with universal prognostic guidance to detect survival markers are still scarce. Preoperative computed tomography (CT) scans can be used to modify the surgical strategy by obtaining extensive information about the patient's tumor before the surgery. Creating a reliable and secure method for evaluating survival and devising treatment plans before surgery remains a major hurdle. The rapid evolution of radiomics is due to advances in science and technology, which have made it possible to convert digital medical images into high‐throughput data. By applying advanced computational analysis to images, radiomics extracts features that cannot be perceived by the naked human eye, thereby improving visual assessment. The application of machine learning techniques to radiomics can lead to good performance in predicting cancer prognosis [12]. The highly precise, dynamic, and non‐invasive techniques offered by radiomics are ideal for personalized medicine. Previous studies have shown that CT imaging attributes have been found to be associated with cancer angiogenesis, metabolism, hypoxia, and microenvironment [13, 14]. Radiomics on CT imaging can provide assistance in the early diagnosis, risk stratification, evaluation of residual lesions, and characterization of tumor heterogeneity in patients with HCC [2]. Despite recent progress in radiomics, there is currently no study utilizing radiomics features to predict the expression levels of LOX and evaluate its prognostic value in the survival analysis of HCC patients.

Our current research is focused on investigating the association between LOX expression levels and the prognosis of HCC patients, utilizing data from the Cancer Genome Atlas (TCGA) and matched patient information from the Cancer Imaging Archive (TCIA). We also aimed to create a radiomics model using CT scans that can predict LOX expression levels, serving as a valuable tool for clinical decision‐making.

## 1 Methods

### 1.1 Baseline Data Sheet of HCC

Medical imaging data (including the clinical and follow‐up data) and the transcriptome sequencing data of patients with HCC were downloaded from TCIA and TCGA databases, respectively. Patients with (1) primary, initially treated HCC; (2) complete follow‐up data; (3) complete clinical and pathological information; and (4) available RNA‐seq data from TCGA were included. Meanwhile, patients from TCIA who had undergone surgical resection, with poor image quality or could not meet the requirements of subsequent analysis were excluded (The brief inclusion/exclusion criteria can be seen in Table S1). These data were collected to discuss the prognostic value of LOX, to construct a radiomics prediction model, and to evaluate the prognostic value of this model. We used the R package “survminer” to calculate the cutoff values of LOX expression based on the minimum *p*‐value approach, and to divide the patients into LOX high‐expression and LOX low‐expression groups.

### 1.2 Difference Analysis of Tumor and Normal Samples

RNA‐seq data from the STAR process of the TCGA‐LIHC project were downloaded and collated from the TCGA database. Data in the fragments per kilobase of exon model per million mapped fragments (FPKM) format were extracted from the collected RNA‐seq data via the Toil process. To eliminate sequencing depth and other systematic biases between samples, a log2 transformation was applied to the RNA‐seq data in FPKM format to make the data more normally distributed and convenient for subsequent statistical comparison. The R packages “ggplot2” (ver. 3.3.6), “stats” (ver. 4.2.1) and “car” were used for visualization.

### 1.3 Survival Analysis

Kaplan–Meier curves were used to demonstrate the survival rate of different groups, and significance tests were made on the survival rate of groups by Log‐rank test. Cox regression was applied in univariate and multivariate analyses of factors that affect OS, and a univariate Cox regression was also used for exploratory subgroup analysis to analyze the impact of LOX expression on the prognosis of patients in each covariate subgroup. A likelihood ratio test was used to analyze the interaction between LOX expression and other covariates. The R package “survival” was used for the survival analysis of each variant, and “survminer” and “forestplot” were used for summarization and visualization.

### 1.4 Analysis of Correlation Between the LOX Expression and Clinical Parameters, Immune‐Related Genes, and Immune‐Cell Infiltration

The gene expression matrix of HCC samples was uploaded to the CIBERSORTx database to calculate the immune‐cell infiltration of each sample. Spearman's rank correlation coefficient was used to analyze the correlation between the LOX expression and clinical parameters, immune‐related genes, and immune‐cell infiltration. Statistical significance was defined as a two‐tailed *p* value of < 0.05, and the *p* values were corrected for multiple comparisons by Bonferroni correction.

### 1.5 Enrichment Analysis of Differently Expressed Genes (DEGs) of the LOX High‐Expression and Low‐Expression Groups

The gene set enrichment analysis (GSEA) was performed to gain insight into the molecular mechanism associated with LOX expression differences, which can ensure activity changes of a predefined gene cluster (KEGG, Hallmark, etc.) between two different types of sample clusters. The DEGs were screened out with a *p*‐value < 0.05 as the significance level using the R package “stats”. Then, we performed the Hallmark and KEGG enrichment analyses in the R package “clusterProfiler”, and visualized the top 20 significantly‐enriched pathways. Statistical significance was defined as a two‐tailed *p* value of < 0.05. *p* values were corrected for multiple comparisons using the false discovery rate (FDR), and an adjusted *p* value (p.adjust) of < 0.05 was used as the significance threshold.

### 1.6 Radiomics Model Construction and Evaluation

We constructed 2 radiomic models based on whole‐tumor area and the combination of whole‐tumor and peri‐tumor area, respectively. We selected the data of patients with HCC in the intersection of TCGA and TCIA databases and divided them into LOX high‐expression and LOX low‐expression groups with cutoff value = 1.1391. In order to reduce the influence of different scanning devices, imaging protocols, and patient differences in lesion size, we adopted the following image preprocessing measures: all raw images were resampled to 1 × 1 × 1 mm^3^ pixels, a bin width = 25 HU was used to discretize the voxel intensity values, the image gray value was normalized to 1–500 HU, and the gray value was standardized by Z‐score. Radiomics features were standardized by the preProcess function from the R package “caret” Radiomics features were extracted from the imaging data of selected patients by R with package “pyradiomics”

The intraclass correlation coefficient (ICC) can measure and evaluate the degree of correlation and agreement between different measurements or observers, which was used to filter effective radiomics features for further analysis. Two experienced radiologists were recruited to delineate the tumor region of each patient to obtain the volume of interest (VOI) in 3D Slicer (ver. 4.10.2) manually. Then, the Python package “SimpleITK” was recruited to delineate the whole‐tumor and peri‐tumor region based on the VOI. Specifically, the whole‐tumor region was extended outwards by 3 mm to obtain a combined region containing the whole tumor and its surrounding region within 3 mm. When one of the radiologists delineated the VOI of all 34 samples, the other radiologist delineated the VOI of 10 samples selected with a random number table method. The radiomics features were extracted from the VOI delineated by radiologists, and the ICC of each feature was calculated using the R package irr. ICC ≥ 0.75 was considered high consistency, 0.51–0.74 as medium, and < 0.5 as low. Extracted radiomics features with an ICC of ≥ 0.75 met the criteria for further analysis, while the others were excluded from the final feature dataset.

The least absolute shrinkage and selection operator (LASSO) was used to select the most useful predictive features. Using the R package “glmnet”, 1000 LASSO regressions were performed on all candidate features. The training set is randomly sampled in each regression, and the optimal regularization parameter *λ* is determined based on cross‐validation. Then, the frequency of each feature being retained in 1000 regressions was counted, and finally, features that emerged > 500 times were selected as stable key features included in the model. The results of the LASSO regression analysis were demonstrated by typical LASSO cross‐validation curves; coefficient path plots showed the process of the feature coefficient from initial to shrinking to 0 as the value of *λ* changed, and histograms showed the frequency of occurrences of the features. Support vector machine (SVM) can acquire a high‐dimensional hyperplane as a decision border by support vectors. We performed SVM modeling on the features selected above to predict gene expression with the R package “caret”. With SVM, we could acquire the parameter Radiomics_score, which could possibly predict the expression of LOX. Wilcoxon test analyses were used for exploring the difference in Radiomics_score between the LOX high‐expression and low‐expression groups, and the results were visualized using the R package “Ggpubr”.

The receiver operating characteristic (ROC) and precision recall (PR) curves were plotted to evaluate the radiomic model. The diagnostic index was calculated by ROC and PR curves including the area under the curve (AUC), accuracy (ACC), sensitivity (SEN), specificity (SPE), positive predictive value (PPV) and negative predictive value (NPV) to evaluate the diagnostic performance. The calibration curves were plotted, and the Hosmer‐Lemeshow test was used to evaluate the calibration of the predictive model. The Brier score was utilized to quantify the comprehensive performance, and a decision curve analysis (DCA) was employed to assess the clinical usefulness of the radiomic model.

An internal 5‐fold cross‐validation was performed to validate the radiomic model. Specifically, the samples were evenly divided into five subsets, which were designated as the validation set in turn, and the remaining subsets were allocated for training. This process was repeated five times to ensure the stability and generalization ability of model evaluation.

**FIGURE 1:**
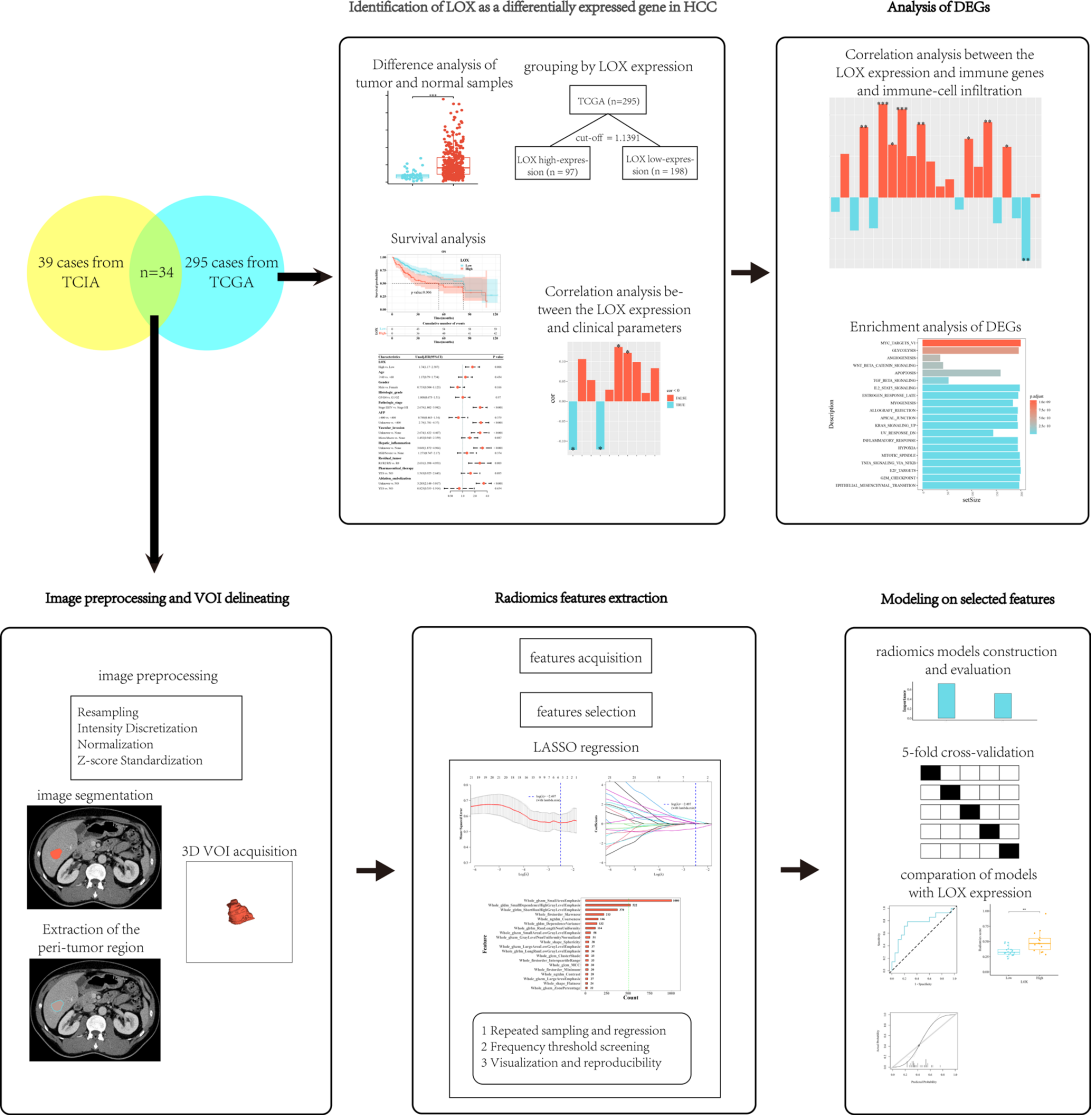
Workflow of the study. DEGs, differently expressed genes; LASSO, the least absolute shrinkage and selection operator; LOX, the lysyl oxidases; TCGA, the Cancer Genome Atlas; TCIA, the Cancer Imaging Archive. *p < 0.05, **p < 0.01, ***p < 0.001.

The AUC values of the two models with the training and validation sets were compared using the DeLong test. The AUC value greater than 0.7 signifies the presence of a high‐performance prediction model [15, 16]. Furthermore, when the difference between the AUC value in the training set and 5‐fold cross‐validation set is less than 0.1, the model is deemed to be non‐significantly overfitted [17]. The workflow of the current study is illustrated in Figure 1.

## 2 Results

### 2.1 Identification of LOX as a Differentially Expressed Gene in HCC

A total of 295 patients with hepatocarcinoma from the TCGA database were included in the survival analyses (Table S1). The R package “survminer” was utilized to calculate the cutoff value of LOX expression level, which was determined to be 1.1391. The patients were subsequently categorized into LOX high‐expression (*n* = 97) and LOX low‐expression groups (*n* = 198), with no significant difference in ages between the two groups (Table 1).

**TABLE 1:** Clinical characteristics of the population with high and low LOX expression group.

Variables	Total (*n* = 295)	Low (*n* = 198)	High (*n* = 97)	*p*
Age, *n* (%)				0.429
< 60	142 (48)	99 (50)	43 (44)	
≥ 60	153 (52)	99 (50)	54 (56)	
Gender, *n* (%)				0.052
Female	92 (31)	54 (27)	38 (39)	
Male	203 (69)	144 (73)	59 (61)	
Histologic_grade, *n* (%)				0.027
G1/G2	183 (62)	132 (67)	51 (53)	
G3/G4	112 (38)	66 (33)	46 (47)	
Pathologic_stage, *n* (%)				0.052
Stage I/II	220 (75)	155 (78)	65 (67)	
Stage III/IV	75 (25)	43 (22)	32 (33)	
AFP, *n* (%)				0.068
< 400	159 (54)	112 (57)	47 (48)	
≥ 400	74 (25)	52 (26)	22 (23)	
Unknown	62 (21)	34 (17)	28 (29)	
Vascular_invasion, *n* (%)				0.335
None	167 (57)	118 (60)	49 (51)	
Unknown	40 (14)	25 (13)	15 (15)	
Micro/Macro	88 (30)	55 (28)	33 (34)	
Hepatic_inflammation, *n* (%)				0.857
None	101 (34)	69 (35)	32 (33)	
Unknown	94 (32)	64 (32)	30 (31)	
Mild/Severe	100 (34)	65 (33)	35 (36)	
Residual_tumor, *n* (%)				0.9
R0	273 (93)	184 (93)	89 (92)	
R1/R2/RX	22 (7)	14 (7)	8 (8)	
Pharmaceutical_therapy, *n* (%)				0.136
No	265 (90)	182 (92)	83 (86)	
Yes	30 (10)	16 (8)	14 (14)	
Ablation_embolization, *n* (%)				0.102
No	224 (76)	143 (72)	81 (84)	
Unknown	50 (17)	39 (20)	11 (11)	
Yes	21 (7)	16 (8)	5 (5)	

**FIGURE 2:**
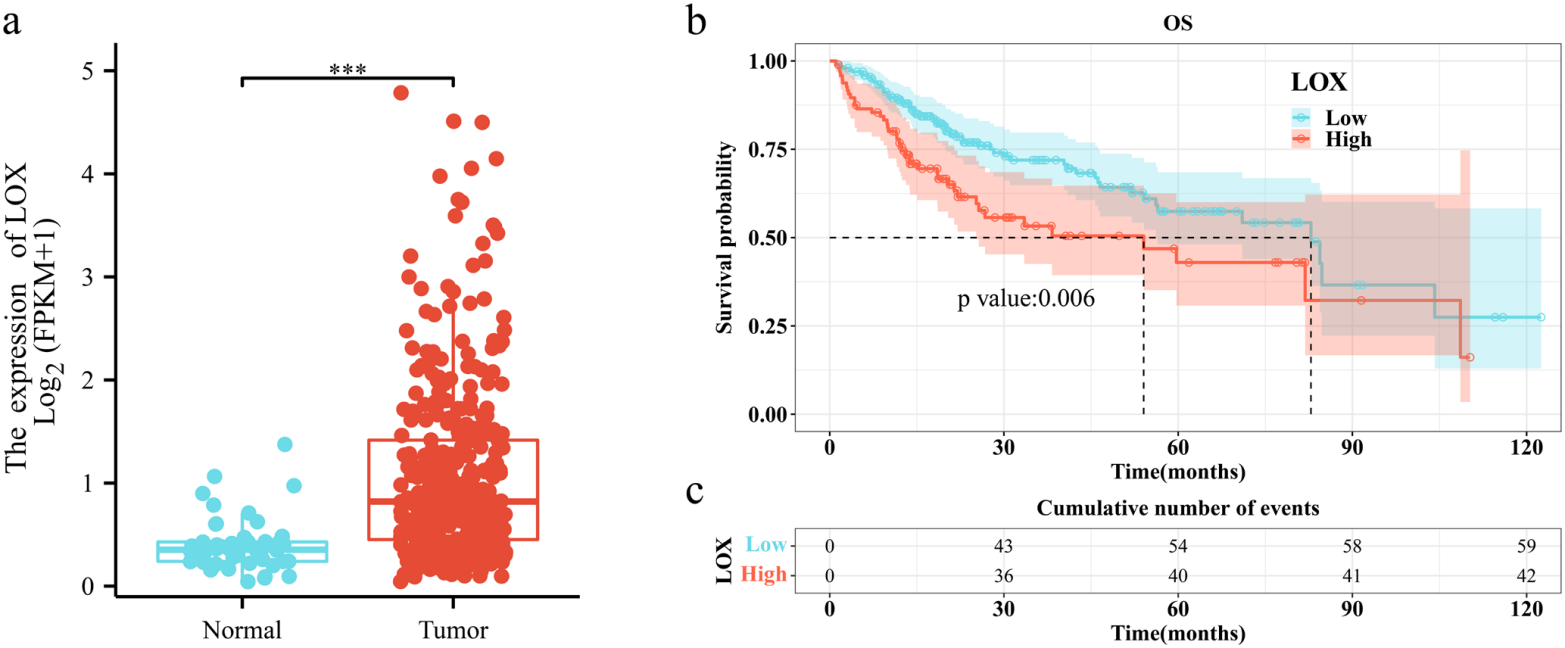
A comparison of the LOX expression level between the normal tissues and HCC tissues and the comparison of survival data. (a) The expression level of LOX in HCC tissues was significantly higher than that in normal tissues. (b) The Kaplan–Meier curve shows the high expression of LOX was significantly correlated with worse OS. (c) The cumulative number of events of LOX low‐expression group and high‐expression group. ***p < 0.001.

**FIGURE 3:**
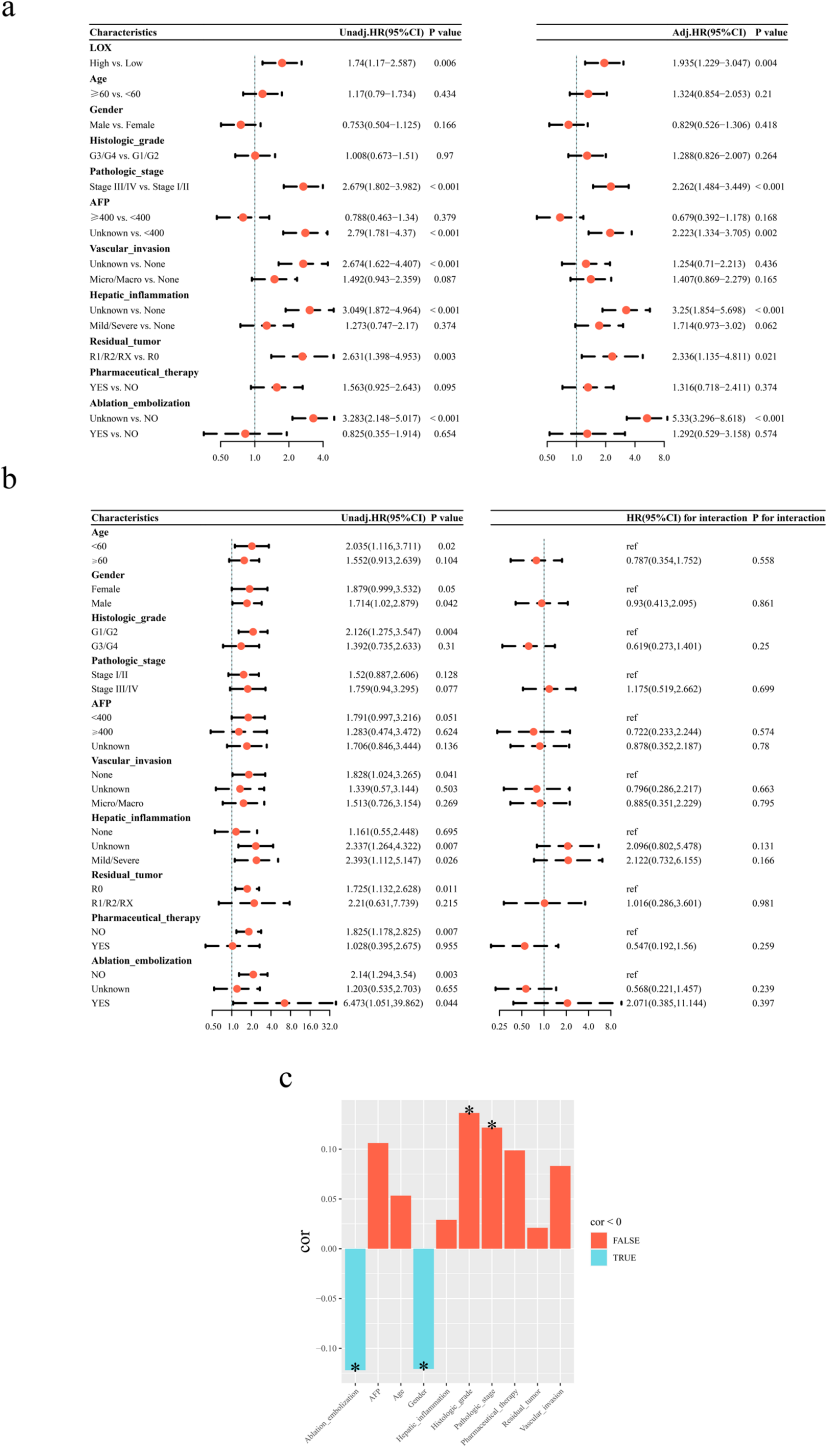
Clinicopathological characteristics in relation to overall survival and LOX expression levels. Univariate Cox regression analysis and multivariate Cox regression analysis revealed that high expression of LOX was a risk factor for HCC, and the result was statistically significant (a). The subgroup analysis also revealed that high LOX expression is a risk factor in patients aged < 60 years, with no significant interaction between age and the expression of LOX in patients with HCC shown in the interaction test (b). The LOX expression showed a significant correlation with ablation embolization, gender, histologic grade, and the pathologic stage (c). Cor, correlation coefficient; *p*. adjust, adjusted *p* value; *p < 0.05.

The expression level of LOX in tumor tissues was significantly higher than that in normal tissues, with a median difference of 0.45469 (95% confidence interval [CI], 0.31836–0.61311, *p* < 0.001) (Figure 2a). The median survival time of the LOX low‐expression group and high‐expression group was 82.87 and 54.07 months, respectively. Kaplan–Meier curves showed the high expression of LOX was significantly correlated with worse OS (*p* < 0.006) (Figure 2b,c). Furthermore, the elevated expression of LOX was identified as a risk factor in both univariate and multivariate Cox regression analyses. Specifically, the univariate analysis yielded a hazard ratio (HR) of 1.74 (95% CI: 1.17–2.587, *p* = 0.006), while the multivariate analysis resulted in an HR of 1.935 (95% CI: 1.229–3.047, *p* = 0.004) (Figure 3a).

The subgroup analysis further elucidated that elevated LOX expression is a risk factor in patients aged < 60 years (HR = 2.035, 95% CI: 1.116–3.711, *p* = 0.02), while demonstrating no significant impact in patients aged ≥ 60 years (HR = 1.552, 95% CI: 0.913–2.639, *p* = 0.104). The interaction test showed that there was no significant interaction between age and the expression of LOX in patients with HCC (*p* = 0.558) (Figure 3b). The LOX expression exhibited a significant correlation with the ablation, embolization, gender, histologic grade, and the pathologic stage (Figure 3c).

### 2.2 Analysis of DEGs in HCC Associated With High and Low Expression of LOX

**FIGURE 4:**
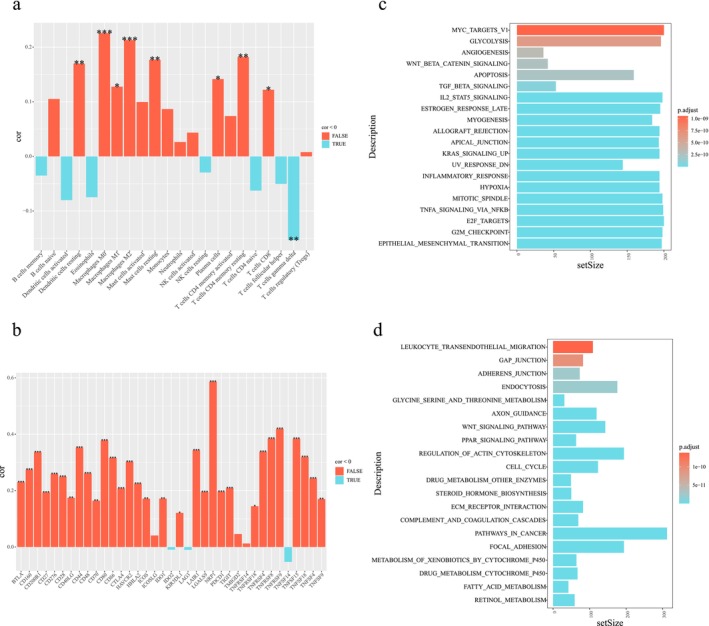
Analysis of infiltration of immune cells and differentially expressed genes (DEGs) in HCC associated with LOX expression. (a) Correlation heatmaps between LOX expression and infiltration of immune cells. (b) Correlation heatmaps between LOX expression and immune genes. (c) Significantly enriched pathways associated with DEGs in HCC related to LOX expression in Hallmark. (d) Significantly enriched pathways associated with DEGs in HCC related to LOX expression in KEGG. Cor, correlation coefficient; SetSize, a variant representing the number of genes contained in a pathway or gene set. *p*. adjust, adjusted *p* value; *p < 0.05, **p < 0.01, ***p < 0.001.

As shown in the heatmap, there was a significant correlation between the macrophages M0 and LOX expression (Figure 4a). Besides, the expression of LOX exhibited a positive correlation with TNFRSF18, TNFSF4, and other genes (*p* < 0.001) (Figure 4b). The statistical significance was maintained even following the most stringent Bonferroni correction (*p* < 0.001 < 0.05/37≈0.00135).

GSEA revealed the top 20 pathways that were significantly enriched, associated with DEGs in HCC related to high and low LOX expression in Hallmark and KEGG. As indicated by the Hallmark enrichment analysis, DEGs were predominantly enriched in the Wnt‐β catenin signaling pathway, the IL2‐STAT5 signaling pathway, and the TGF‐β signaling pathway. By contrast, the KEGG enrichment analysis indicated that the DEGs were enriched in the Wnt signaling pathway and the PPAR signaling pathway (Figure 4c,d).

### 2.3 Construction and Evaluation of HCC Radiomics Models

**FIGURE 5:**
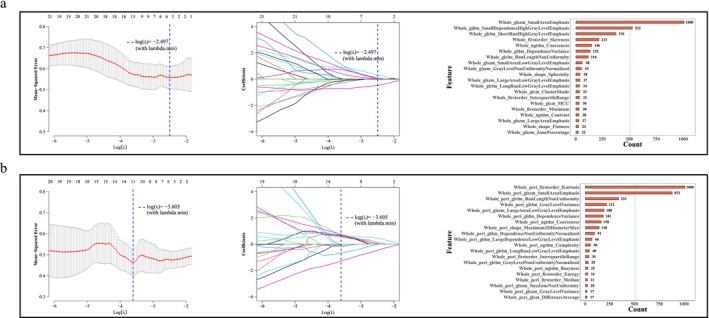
Features were selected by LASSO based on whole‐tumor region (a) and the combination of whole‐tumor and peri‐tumor regions (b).

A total of 34 HCC patients from the intersection of TCGA and TCIA database TCIA‐CT databank were enrolled. Based on whole‐tumor region, 107 radiomic features were enrolled, and 91 of them with ICC ≥ 0.75 were selected for further analysis after filtration. Figure 5 demonstrated LASSO paths and the visualization of feature selection. The left and middle parts of Figure 5a,b were typical LASSO cross‐validation curves and coefficient path maps, and the right part showed the frequency of occurrences of the features based on the results obtained by repeated LASSO. The features of most counts were plotted in a histogram, and the following features were selected by LASSO: Whole_glszm_SmallAreaEmphasis and Whole_gldm_SmallDependenceHighGrayLevelEmphasis (Figure 5a). Based on the combination of whole‐tumor and peri‐tumor region, 107 radiomic features were enrolled, of which 99 exhibited an ICC ≥ 0.75 and were selected for subsequent analysis after filtration. The features of most counts were plotted in a histogram, and two features were selected by LASSO: Whole_peri_firstorder_Kurtosis and Whole_peri_glszm_SmallAreaEmphasis (Figure 5b). The parameters of ICC values of radiomic features were summarized in Table S2, and the importances of selected features acquired by SVM were shown in Figure S1.

**FIGURE 6:**
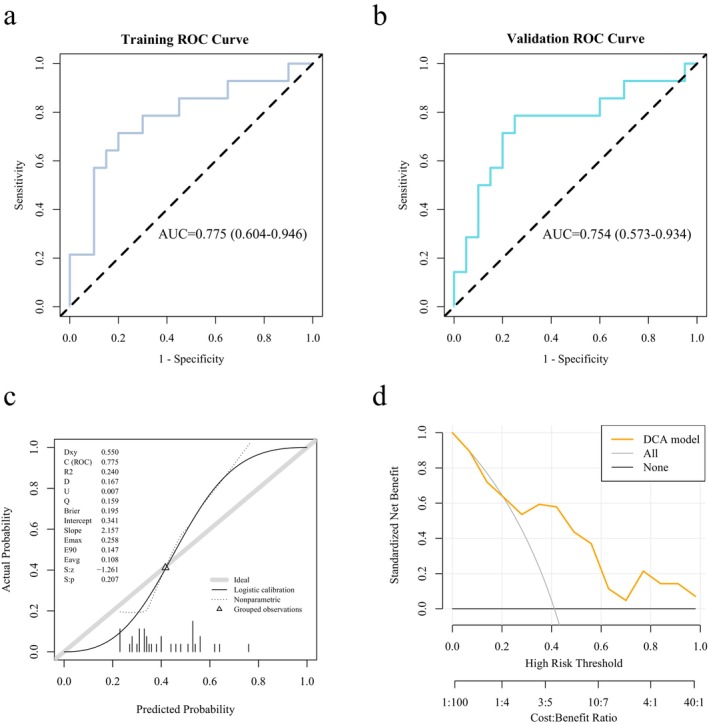
The performance of the radiomics model of the whole‐tumor region for predicting the LOX expression level with 5‐fold cross‐validation on the training and validation set. (a) ROC curves of the model. AUC = 0.775 (0.604–0.946), threshold = 0.425, accuracy = 0.765, sensitivity = 0.714, specificity = 0.8, and Brier score = 0.195. (b) ROC curves of 5‐fold cross‐validation of the model. AUC = 0.754 (0.573–0.934), threshold = 0.37, accuracy = 0.765, sensitivity = 0.786, specificity = 0.75, and Brier score = 0.2. (c) Calibration curves of the model. (d) DCA curve for the model. The model (yellow line) received a higher net benefit than all treatments (gray line) and no treatment (black line) with a threshold probability of 0.21–1.0.

The radiomic model of the whole‐tumor region has a good predicting effect in the expression of LOX. The AUC value of the ROC curve of the radiomics model of the whole‐tumor region was 0.775 in the training set (95% CI: 0.604–0.946). Moreover, the employment of 5‐fold cross‐validation in the evaluation of the model yielded an average AUC value of 0.754 (95% CI: 0.573–0.934), indicating that the prediction performance of the model in the internal validation was stable (Figure 6a,b). The discrepancy between the AUC value in the training set and the 5‐fold cross‐validation sets was 0.021, indicating that the model did not exhibit signs of overfitting. The calibration curves (Figure 6c) and the Hosmer‐Lemeshow test revealed the consistency of the prediction probability obtained by our radiomic model and the true value of LOX expression (*p* = 0.64 > 0.05). As shown by the DCA curve, the model achieved the maximum net benefit with a threshold probability of 0.21–1.0, as compared with all treatments and no treatment, indicating that the model had clinical practicability (Figure 6d). In the training set, the threshold was 0.425; the accuracy, sensitivity, specificity, and Brier score were 0.765, 0.714, 0.8, and 0.195, respectively; in the 5‐fold cross‐validation cohort, the threshold was 0.37; the accuracy, sensitivity, specificity, and Brier score were 0.765, 0.786, 0.75, and 0.2, respectively (Table S3).

**FIGURE 7:**
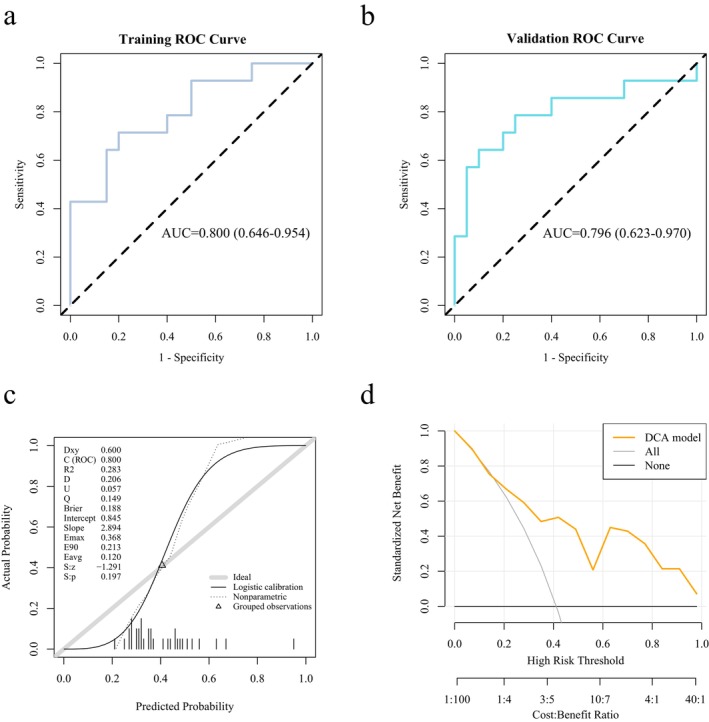
The performance of the radiomics model of the whole‐tumor and peri‐tumor region for predicting the LOX expression level with 5‐fold cross‐validation on the training and validation set. (a) ROC curve of the model. AUC = 0.8 (0.646–0.954), threshold = 0.397, accuracy = 0.765, sensitivity = 0.714, specificity = 0.8, and Brier score = 0.188. (b) ROC curve of 5‐fold cross‐validation of the model. AUC = 0.796 (0.623–0.97), threshold = 0.474, accuracy = 0.794, sensitivity = 0.643, specificity = 0.9, and Brier score = 0.185. (c) Calibration curves of the model. (d) DCA curve for the model. The model (yellow line) received a higher net benefit than all treatments (gray line) and no treatment (black line) with a threshold probability of 0.15–1.0.

The radiomic model of the whole‐tumor and peri‐tumor region also has a good predicting effect in the expression of LOX. The AUC value of the ROC curve for the radiomics model of the whole‐tumor and peri‐tumor region was 0.800 in the training set (95% CI: 0.646–0.954), and the average AUC value obtained from the 5‐fold cross‐validation was 0.796 (95% CI: 0.623–0.970), indicating that the model demonstrated stable prediction performance in the internal validation (Figure 7a,b). The discrepancy between the AUC value in the training set and the 5‐fold cross‐validation sets was 0.004, suggesting that the model did not exhibit signs of overfitting. The calibration curves (Figure 7c) and the Hosmer‐Lemeshow test revealed the consistency of the prediction probability acquired by our radiomic model and the true value of LOX expression (*p* = 0.36 > 0.05). As shown by the DCA curve, the model achieved the maximum net benefit with a threshold probability of 0.15–1.0 compared with all treatments and no treatment, indicating that the model had clinical practicability (Figure 7d). In the training set, the threshold was 0.397; the accuracy, sensitivity, specificity, and Brier score were 0.765, 0.714, 0.8, and 0.188, respectively; in the 5‐fold cross‐validation cohort, the threshold was 0.474; the accuracy, sensitivity, specificity, and Brier score were 0.794, 0.643, 0.9, and 0.185, respectively (Table S4).

**FIGURE 8:**
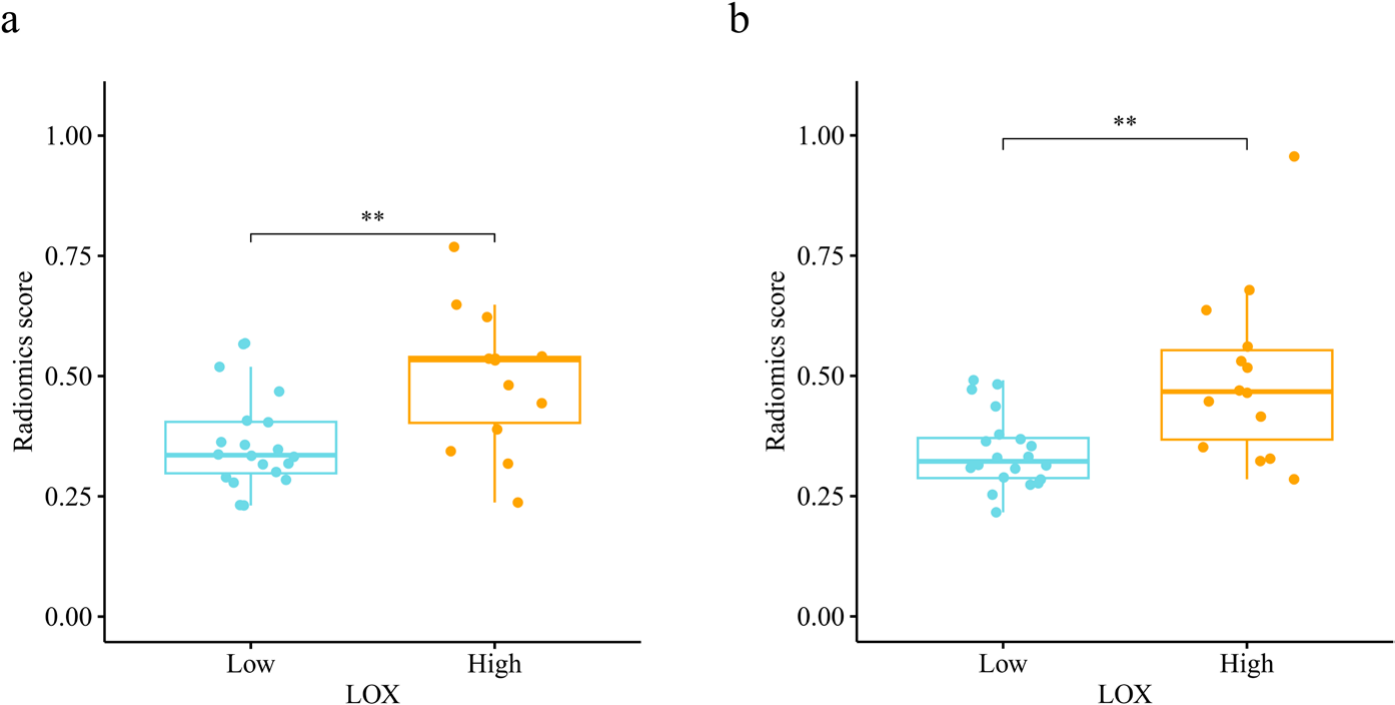
Correlation of radiomics score and LOX expression in the radiomics model of whole‐tumor (a) and whole‐tumor and peri‐tumor region (b). **p < 0.01.

In addition, the Radiomics_score of the LOX high‐expression group was significantly higher than the low‐expression group in both of the radiomic models, with a *p* value of 0.0062 in the radiomic model of whole‐tumor and 0.0062 in the radiomic model of whole‐tumor and peri‐tumor (Figure 8).

The AUC of the radiomic model of the whole‐tumor and peri‐tumor region was slightly higher than the model of the whole‐tumor region. The DeLong test showed no statistical significance between the two models, with a *p* value of 0.724 (95% CI: 0.646–0.954) in the training cluster and 0.729 (95% CI: 0.623–0.970) in the cross‐validation cluster.

## 3 Discussion

Primary liver cancer is the fourth leading cause of cancer‐related deaths worldwide, with significant histological and biological heterogeneity. The survival rate of patients varies widely [18]. Identifying genes that help predict HCC outcomes could facilitate personalized treatment and management strategies. LOX, a secreted copper‐dependent amine oxidase, has been identified in a variety of studies. Aberrant expression or activity of the LOX family occurs in various types of cancer [7]. It has been proved that the LOX family mainly performs tumor microenvironment (TME) remodeling functions and is extensively involved in tumor invasion and metastasis, immunomodulation, proliferation, apoptosis, etc. [5] Several studies have indicated a potential correlation between LOX and prognosis in HCC [19], despite the paucity of research on the subject. However, the role of LOX in the development of HCC remains unclear. In order to draw reliable conclusions, the bioinformatics data from the TCGA was analyzed. In our study, the level of LOX expression was found to be significantly elevated in HCC tissues compared to normal tissues. LOX has the ability to distinguish between normal tissue and tumors based on the ROC curve. Meanwhile, the macrophages M0 exhibited a substantial correlation with LOX expression. Besides, the expression of LOX was positively associated with several immune genes, including TNFRSF18, TNFSF4, and other immune genes. These findings indicated that LOX may participate in the immune infiltration process of HCC and play a certain role. In summary, LOX may facilitate the identification and diagnosis of HCC.

As the classical approaches for HCC prognosis, such as CT, B‐ultrasound, and PET‐CT, do not fulfill the demands of precision medicine [20], it is crucial to pinpoint fresh prognostic indicators and stratify patients based on their prognosis, thereby enabling customized precision treatment. Novel prognostic markers and patient stratification based on prognosis are vital in providing new indicators for personalized precision therapy. The acquisition of further molecular information through radiomics analysis of CT images could provide a new strategy for therapeutic decision‐making [21]. The purpose of this investigation was to explore the connections between the radiomics profile of HCC and both LOX expression levels and the patient's overall survival rate.

Molecular markers, molecular prediction of prognosis, bioinformatics, machine learning, and artificial intelligence technologies are being applied to cancer research. According to the findings of Cheng et al., a radiomics model containing 102 selected features showed an encouraging discrimination performance of epidermal growth factor receptor (EGFR) mutation status (mutant or wild type), and the predictive ability of the model was superior to that of the clinical model (AUC: 0.838 vs. 0.674, 0.822 vs. 0.730, and 0.803 vs. 0.746) for the training, internal validation, and external validation sets, respectively [22]. Xiao et al. applied a radiomics model to predict SYP gene expression, which the prediction model yielded an accuracy of 0.93 [23]. According to Peng et al., prediction is feasible using CT radiomics, achieving an AUROC of 0.914 when combined with clinical and CT semantic features [24]. Furthermore, 5‐fold cross‐validation has been identified as an effective internal validation method for evaluating the performance of radiomics models in the fields of medical imaging and machine learning [25]. In our study, after features screening based on repeat LASSO using the optimal lambda of 107 in the whole‐tumor region, two optimal features: Whole_glszm_SmallAreaEmphasis and Whole_gldm_SmallDependenceHighGrayLevelEmphasis were selected for constructing the model. The predictive performance of models predicting LOX expression levels was good. The AUC value of the ROC curve of the radiomics model of the whole‐tumor region was 0.775 in the training set, and the average AUC value obtained from the 5‐fold cross‐validation was 0.754, indicating the stable prediction performance of the model in the internal validation. The discrepancy between the AUC value in the training set and the 5‐fold cross‐validation sets was 0.021, which was significantly less than 0.1, prompting the model did not exhibit signs of overfitting. The calibration curves and the Hosmer‐Lemeshow test revealed the consistency of the prediction probability acquired by our radiomic model and true value of LOX expression (*p* = 0.64 > 0.05). The DCA curve showed that the model exhibited clinical practicability. The findings revealed that the amalgamation of clinical and radiomics models improved model performance, thereby suggesting that utilizing both types of models can be beneficial.

Peritumoral tumor refers to the boundary area between tumor entity and healthy tissue. Studies have shown that peritumoral tissue behaves the same as normal tissue on a macro level, but actually has microscopic heterogeneity [26]. For a long time, most studies have focused on the study of tumor tissue. With the deepening of the study of tumor microenvironment (TME), Gu et al. proposed the concept of peritumor microenvironment (PME) for the first time, and confirmed through research that PME of liver cancer has a unique microenvironment, which is involved in the whole process of tumor occurrence and development, and is of great significance for tumor diagnosis and treatment [27]. Genetic, epigenetic, and transcriptomic alterations have been identified in paracancer tissues of many epithelial cancers, including cancers of head and neck, colorectal, skin, bladder, lung, prostate, ovary, and breast [28]. However, the heterogeneity of normal peritumoral tissue in gross morphology is difficult to assess during routine preoperative examination. Based on the previous imaging omics research on the interior of tumors, the concept of peritumoral imaging research has gradually been applied to all systemic diseases affecting the entire body. A large number of research results show that the peritumoral imaging model also shows a good diagnostic and predictive effect on the diagnosis and prediction of tumors, and can be used as an important supplementary means. A number of studies even found that the peritumoral omics model exhibits superior predictive value in comparison to the intratumoral model [29, 30]. Wu et al. developed imaging labels based on the intratumoral and circumumoral 10‐mm imaging features of the automatic full‐volume ultrasound images of the breast, combined with the supertumor size and lymph node status to construct a comprehensive nomogram to predict the expression of Ki‐67, demonstrating the AUC of the training set was 0.905 and the AUC of the verification set was 0.882, and the diagnostic specificity in both the training set and the verification sets was over 90.9% [31]. Jiang et al. extracted peritumoral 4‐mm omics characteristics based on a multimodal study of mammography and MRI, and also obtained similar results as above [32]. In the study of EGFR mutation in lung cancer, Yamazaki et al. analyzed the 3‐mm imaging features of CT images of 478 cases of primary lung cancer and found that combined intratumoral and peritumoral imaging models showed improved efficacy in predicting EGFR mutations [33]. In our study, the radiomic model of the whole‐tumor and peri‐tumor region also has good predicting effect in the expression of LOX. The AUC value of the ROC curve for the radiomics model of the whole‐tumor and peri‐tumor region was 0.800 in the training set, and the average AUC value obtained from the 5‐fold cross‐validation was 0.796, indicating that the prediction performance of the model in the internal validation was stable. The difference between the AUC value in the training set and the 5‐fold cross‐validation sets was 0.004, prompted the model of the whole‐tumor and the peri‐tumor region did not exhibit signs of overfitting. The calibration curves and the Hosmer‐Lemeshow test revealed the consistency of the prediction probability acquired by our radiomic model and the true value of LOX expression (*p* = 0.36 > 0.05). The DCA curve showed that the model exhibited clinical practicability. In the training set, the threshold was 0.397, the accuracy, sensitivity, specificity, and Brier score were 0.765, 0.714, 0.8, and 0.188, respectively; in the 5‐fold cross‐validation cohort, the threshold was 0.474, the accuracy, sensitivity, specificity, and Brier score were 0.794, 0.643, 0.9, and 0.185, respectively. In addition, the Radiomics_score of the LOX high‐expression group exhibited a significant increase compared to the low‐expression group in both radiomic models, with a *p* value of 0.0062 in the radiomic model of the whole‐tumor and 0.0062 in the radiomic model of the whole‐tumor and peri‐tumor. The AUC of the radiomic model of the whole‐tumor and peri‐tumor region was slightly higher than the model of whole‐tumor region. The DeLong test showed no statistical significance between the two models, with a *p* value of 0.724 in the training cluster and 0.729 in the cross‐validation cluster. We observed a slightly higher AUC in the whole‐tumor and peri‐tumor model compared to the whole‐tumor model, which may imply some potential advantages of integrating peritumoral information in clinical risk stratification and prognostic evaluation. In the aforementioned studies based on peritumoral imaging, most of the combined intratumoral and peritumoral models showed the best predictive power, indicating that peritumoral tissues contain important information for tumor genetic prediction.

It is important to acknowledge the potential for a correlation between the selected imaging features and tumor progression, tumor microenvironment, or LOX expression. In our study, Whole_glszm_SmallAreaEmphasis and Whole_gldm_SmallDependenceHighGrayLevelEmphasis were selected based on the whole‐tumor region, while Whole_peri_firstorder_Kurtosis and Whole_peri_glszm_SmallAreaEmphasis were selected based on the combination of the whole‐tumor and peri‐tumor regions. These imaging features involved concepts of gray level size zone matrix (GLSZM) features, which quantify gray level zones in an image, including first‐order statistics, kurtosis, small area emphasis (SAE) and small dependence high gray level emphasis (SDHGLE) (https://pyradiomics.readthedocs.io/en/latest/features.html#). First‐order statistics describe the distribution of voxel intensities within the image region defined by the mask through commonly used and basic metrics. In our study, first‐order statistics were used to describe the distribution of voxel intensities within the whole‐tumor and peri‐tumor regions. Kurtosis is a measure of the “peakedness” of the distribution of values in the image region‐of‐interest (ROI). A higher kurtosis implies that the mass of the distribution is concentrated towards the tail(s) rather than towards the mean, which may be related to the more heterogeneous nature of the tumor, indicating the progression of the tumor [34]. Small area emphasis (SAE) is a measure of the distribution of small size zones in an image; the greater the value, the more it indicates smaller size zones and more exquisite texture. Small dependence high gray level emphasis (SDHGLE) is a measure of the joint distribution of small dependence with higher gray‐level values in an image. However, most of the above explanations can only be investigated through the use of animal models, and the latest articles have also proven that the radiomics model has cross‐species similarity [35]. In addition, some studies have explored the causal relationship between radiomics features and their corresponding molecules by intervening molecular expression [36, 37]. In HCC, radiomics features were regarded as macroscopic manifestations of changes at the microscopic level [38]. As a key extracellular matrix remodeling factor, LOX expression is likely to constitute the biological basis behind specific imaging texture features, such as SAE and SDHGLE. However, the causal association between radiomics features and LOX expression still needs to be further verified in vivo such as by regulating or knocking down LOX expression to observe its specific effects on imaging phenotypes. Therefore, we will also design relevant animal models to carry out related work in the future. In summary, the imaging features described herein indirectly reveal the complexity of the tumor microenvironment by reflecting the heterogeneity of tumor texture and tissue structure and provide a reasonable biological explanation and theoretical support for further exploration of the correlation between imaging features and LOX expression.

It is necessary to recognize the limitation of our research. Firstly, the limited sample size of the study may have limited the power to detect significant effects; further research with a larger sample size is needed. Secondly, the conclusions drawn from this study are limited by the fact that all data were obtained from the public datasets TCGA and TCIA, which may not accurately reflect the population of interest, other datasets, or imaging protocols. Thirdly, the introduction of an independent external cohort for validation was precluded by the limited number of samples with paired high‐quality imaging data and gene expression information in public databases, which raised concerns about overfitting. Hence, the presence of overfitting in the model was assessed by calculating the difference between the AUC value in the training set and the 5‐fold cross‐validation sets. Moreover, we are actively constructing a prospective imaging cohort and intend to collect fresh HCC tissue to detect LOX expression level for subsequent model validation. This is expected to be progressively advanced in the subsequent stage of research to further enhance the clinical application value and external generalization of the model. Fourthly, the implementation of manual ROI delineation under the supervision of two radiologists may have introduced some inter‐observer variability and affected the reproducibility of the results. Fifthly, image reconstruction algorithms, preprocessing methods, individual differences, and feature extraction algorithms can affect the stability and repeatability of the radiomics features. Sixthly, this study did not introduce stratified cross‐validation, which has been demonstrated to further improve the robustness of model evaluation in studies with small sample size or imbalanced category distribution. The overall distribution of LOX high‐expression and low‐expression groups was balanced, thereby indicating that the absence of a stratification strategy did not materially bias the results in a study that was still in the exploratory stage. Subsequent studies will further optimize the cross‐validation strategy and incorporate stratified cross‐validation to the greatest extent possible to enhance the consistency of model performance under different data structures. Despite its limitations, the study contributes to our understanding of our radiomics‐based prediction model, which exhibited favorable efficiency in predicting LOX gene expression and potentially offered substantial value in guiding clinical prognosis prediction.

## 4 Conclusion

In conclusion, our results suggest that differences in LOX expression levels are associated with the overall survival of liver cancer. We constructed two models of whole tumor and whole tumor & peritumoral radiomics that can effectively predict the expression levels of LOX. Our model therefore has the potential to be widely used as a practical tool for the noninvasive characterization of tumors. It helps to stratify the prognosis and has the potential to help the subsequent development of precision medicine.

## Author Contributions

All authors contributed to the study conception and design. Material preparation and data collection were performed by Kexin Gao, Musa Yaermaimaiti, and Yiwei Wang, and data analysis were performed by Guanggai Xia and Ting Xu. The first draft of the manuscript was written by Kexin Gao and Hongcheng Wang, and all authors commented on previous versions of the manuscript. All authors read and approved the final manuscript.

## Ethics Statement

The authors have nothing to report.

## Consent

The authors have nothing to report.

## Conflicts of Interest

The authors declare no conflicts of interest.

## Supporting information


**Figure S1:** The importances of selected features acquired by SVM in in the radiomic model of whole‐tumor region (a) and whole‐tumor and peri‐tumor region (b).

**Table S1:** Inclusion/exclusion criteria applied to determine the study samples.

**Table S2:** The parameters of ICC values of radiomic features in the radiomic models.

**Table S3:** Parameters of the training set of the radiomics model of whole‐tumor region.

**Table S4:** Parameters of the training set of the radiomics model of whole‐tumor and peri‐tumor region.

## Data Availability

The datasets generated and analyzed during the current study are available in the TCIA repository (https://www.cancerimagingarchive.net/) and TCGA repository (https://portal.gdc.cancer.gov/).
