# Supplementary figures and images for: Bi‐Regional Machine Learning Radiomics Based on CT Noninvasively Predicts LOX Expression Level and Overall Survival in Hepatocellular Carcinoma
Source: Cancer Med. 2025 Aug 12;14(15):e71154. doi: 10.1002/cam4.71154 (PMC12340542; doi:10.1002/cam4.71154)

a

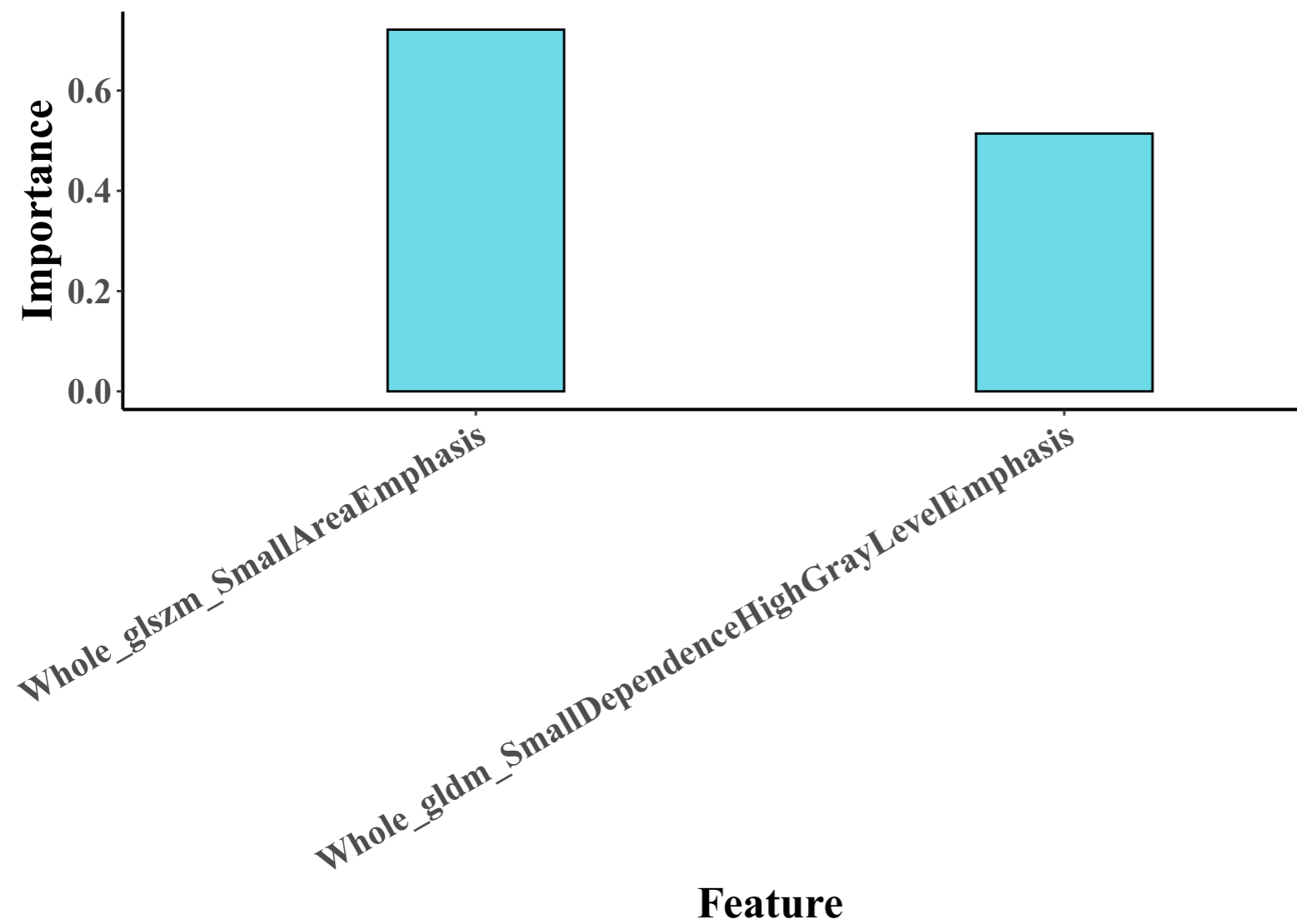

b

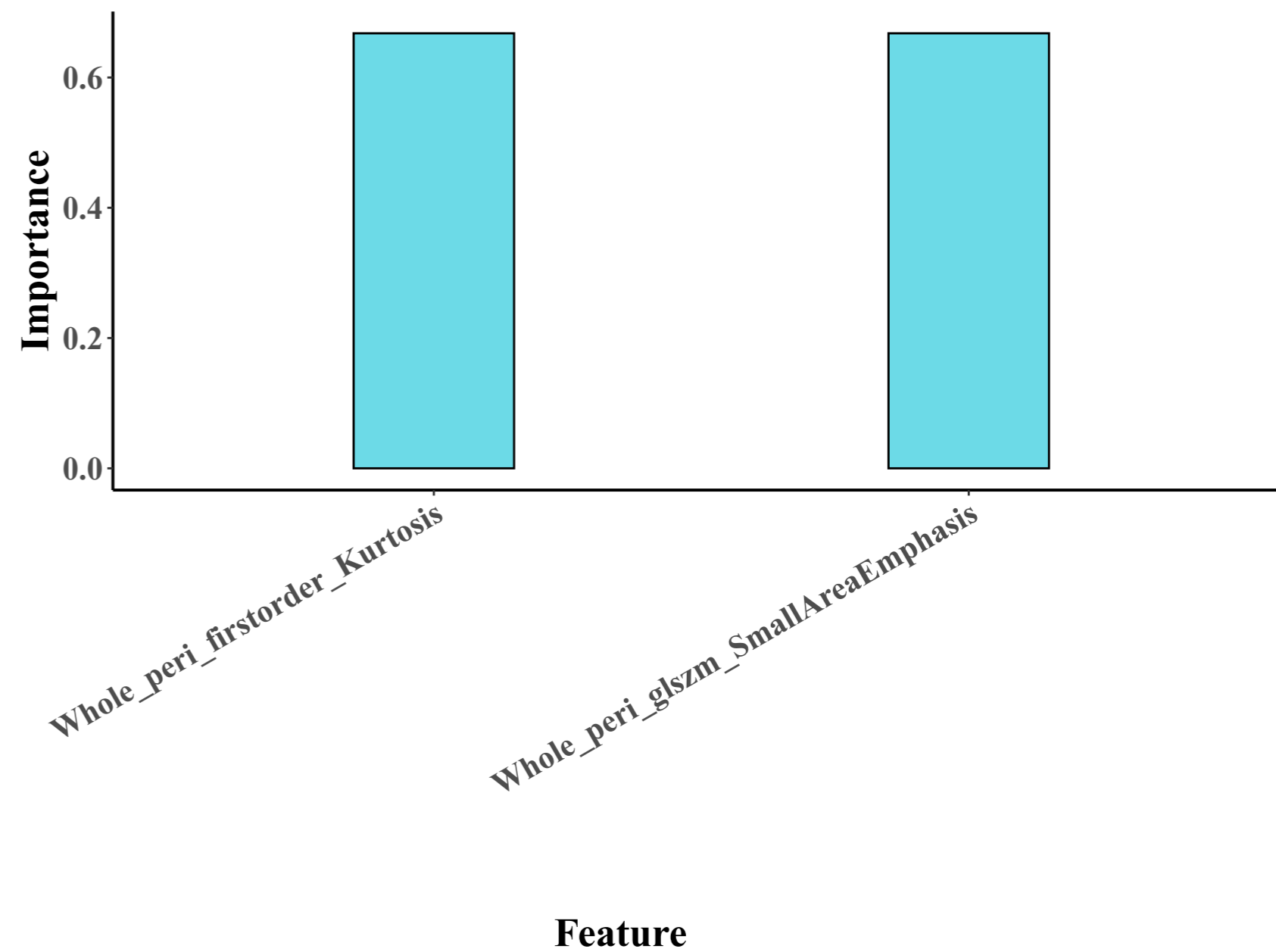

Supplement: Supplementary file 1 — Figure S1: The importances of selected features acquired by SVM in in the radiomic model of whole‐tumor region (a) and whole‐tumor and peri‐tumor region (b). [file CAM4-14-e71154-s001.pdf]
