# Supplementary material for: Bi‐Regional Machine Learning Radiomics Based on CT Noninvasively Predicts LOX Expression Level and Overall Survival in Hepatocellular Carcinoma
Source: Cancer Med. 2025 Aug 12;14(15):e71154. doi: 10.1002/cam4.71154 (PMC12340542; doi:10.1002/cam4.71154)
Supplement: Supplementary file 2 — Table S1: Inclusion/exclusion criteria applied to determine the study samples. [file CAM4-14-e71154-s004.docx]

**Supplemental Table 1**: Inclusion/exclusion criteria applied to determine the study samples

| **TCGA-LIHC clinical data** | Excluded samples | Remaining samples |
| --- | --- | --- |
| Total samples | / | 377 |
| Screening: primary Hepatocellular cancer | 18 | 359 |
| Excluded: OS=NA OS＜30 | 26 | 333 |
| Excluded: Tumor stage grade or residual tumor status unknown | 10 | 343 |
| Screening: primary solid tumor samples with RNA-seq | 8 | **295** |
| **TCIA- LIHC Portal phase enhanced CT data** |  |  |
| Total samples | / | 75 |
| Excluded image: post-operation, poor quality | 36 | 39 |
| Intersection of TCGA-LIHC genomic data | 5 | **34** |

CT: computed tomography; OS: overall survival; TCIA: Cancer Imaging Archive; TCGA: The Cancer Genome Atlas
