# Supplementary material for: Bi‐Regional Machine Learning Radiomics Based on CT Noninvasively Predicts LOX Expression Level and Overall Survival in Hepatocellular Carcinoma
Source: Cancer Med. 2025 Aug 12;14(15):e71154. doi: 10.1002/cam4.71154 (PMC12340542; doi:10.1002/cam4.71154)
Supplement: Supplementary file 3 — Table S2: The parameters of ICC values of radiomic features in the radiomic models. [file CAM4-14-e71154-s003.docx]

**Supplemental Table 2.** The parameters of ICC values of radiomic features in the radiomic models.

| **The parameters of ICC values of radiomic features in the radiomic model of whole-tumor region** | | | | | | |
| --- | --- | --- | --- | --- | --- | --- |
|  | ICC≥0.75 | 0.5≤ICC<0.75 | ICC<0.5 | ICC_Mean | ICC_Median | |
| Percentage | 0.85 | 0.075 | 0.075 | 0.882 | 0.979 | |
| Number | 91 | 8 | 8 | NA | NA | |
| **The parameters of ICC values of radiomic features in the radiomic model of whole-tumor and peri-tumor region** | | | | | | |
|  | ICC≥0.75 | 0.5≤ICC<0.75 | ICC<0.5 | ICC_Mean | ICC_Median |  |
| Percentage | 0.925 | 0.028 | 0.047 | 0.934 | 0.981 |  |
| Number | 99 | 3 | 5 | NA | NA |  |
