# Supplementary material for: Bi‐Regional Machine Learning Radiomics Based on CT Noninvasively Predicts LOX Expression Level and Overall Survival in Hepatocellular Carcinoma
Source: Cancer Med. 2025 Aug 12;14(15):e71154. doi: 10.1002/cam4.71154 (PMC12340542; doi:10.1002/cam4.71154)
Supplement: Supplementary file 5 — Table S4: Parameters of the training set of the radiomics model of whole‐tumor and peri‐tumor region. [file CAM4-14-e71154-s002.docx]

**Supplemental Table 4.** Parameters of the training set of the radiomics model of whole-tumor and peri-tumor region.

| **Parameters of the training set of the radiomics model of whole-tumor and peri-tumor region** | | | | | |
| --- | --- | --- | --- | --- | --- |
| AUC value | threshold | accuracy | sensitivity | specificity | Brier Score |
| 0.8 | 0.397 | 0.765 | 0.714 | 0.8 | 0.188 |
| **Parameters of the validation set of the radiomics model of whole-tumor and peri-tumor region** | | | | | |
| AUC value | threshold | accuracy | sensitivity | specificity | Brier Score |
| 0.796 | 0.474 | 0.794 | 0.643 | 0.9 | 0.185 |
